# The correlation between serum AMH and HOMA-IR among PCOS phenotypes

**DOI:** 10.1186/s13104-018-3207-y

**Published:** 2018-02-09

**Authors:** Budi Wiweko, Indra Indra, Cynthia Susanto, Muharam Natadisastra, Andon Hestiantoro

**Affiliations:** Division of Reproductive Endocrinology and Infertility, Department of Obstetric and Gynecology Faculty of Medicine, Universitas Indonesia; Dr, Cipto Mangunkusumo General Hospital, Jakarta, Indonesia

**Keywords:** Anti-mullerian hormone, Insulin resistance, HOMA-IR, Phenotype, Polycystic ovarian syndrome

## Abstract

**Background:**

Polycystic ovarian syndrome (PCOS) is known to be one of the most prevalent endocrine disorders affecting reproductive age women. One of the endocrine disorder is hyperinsulinemia, which corresponds with the severity of PCOS. However, the pathogenesis of PCOS is not fully understood, but one theory of anti-mullerian hormone (AMH) has been proposed as one of the factor related to the degree of severity of PCOS. However, there are no clear correlation between levels of AMH with the incidence of insulin resistance in PCOS patients especially in Indonesia.

**Methods:**

This is a cross-sectional study involving reproductive age women aged 18–35 years. Subjects were recruited consecutively at Dr. Cipto Mangunkusumo General Hospital between 2011 until 2014. PCOS women diagnosed using 2003 Rotterdam criteria were categorized into four different PCOS phenotypes. Subsequently, serum level of AMH and HOMA-IR was measured and evaluated with correlation tests performed using SPSS 11.0

**Results:**

A total of 125 PCOS patients were included in a study conducted within a 3-year period. Phenotype 1 (anovulation, hyperandrogenism, and polycystic ovaries) shows the highest levels of AMH and HOMA-IR, which decreases in accordance to severity level (p < 0.005). The positive correlation between AMH and HOMA-IR persisted even after adjusting for BMI in multivariate analysis.

**Conclusions:**

There was a positive correlation between serum AMH and HOMA IR levels. Serum AMH and HOMA IR levels were significantly different across the four PCOS phenotypes; with the highest values were present with phenotype 1.

## Background

Polycystic ovarian syndrome (PCOS) is known to be one of the most prevalent endocrine disorders affecting 15–20% of reproductive age women and is a primary cause of infertility [1, 2]. The clinical features of PCOS include menstrual irregularity, chronic anovulation, infertility, and hyperandrogenism [3, 4]. Hormonal imbalance in PCOS manifests as hyperandrogenism and hyperinsulinemia, with reciprocal negative effects and this corresponds with the severity of PCOS. Hyperinsulinemia could increase androgen and free androgen production by reducing the binding of androgen with sex-hormone binding globulin (SHBG). These hormonal imbalances could progress to metabolic and cardiovascular diseases. In a previous cohort study, abnormalities in glucose metabolism were present for 65% of the cardiovascular disease-related deaths [5].

To date, the pathogenesis of PCOS is not fully understood. One of the proposed mechanism for hyperandrogenism is follicle maturation abnormalities, in which the growing follicle does not progress to a dominant follicle. Follicle maturation depends on the levels of follicle stimulating hormone (FSH), which are reportedly suppressed to a level below the threshold for aromatase enzyme activation in patients with PCOS, resulting in high androgen levels. Another proposed pathology related to follicle maturation abnormality is reduced follicle sensitivity to FSH stimulation by anti-mullerian hormone (AMH) [6, 7]. AMH is a dimeric glycoprotein secreted by the granulose cells, and a member of the transforming growth factor-β (TGF-β) family [8, 9]. In PCOS, levels of AMH are found 3 times higher than in healthy woman [8]. In Indonesia, level of AMH is a useful marker with high sensitivity and specificity for predicting PCOS [10]. The exact mechanism responsible for high level of AMH in PCOS is still poorly understood. Studies concluded that obesity, insulin resistance, and hyperandrogenism play major roles in the increasing level of AMH [8, 11, 12].

AMH level was recently found to be related to the degree of severity of PCOS [10, 11]. The severity of PCOS was divided into categories based on patients’ phenotypes. According to the Rotterdam criteria (2003) [13], there are four known phenotypes of PCOS, namely: (1) phenotype 1, characterized by anovulation, hyperandrogenism, and polycystic ovaries; (2) phenotype 2, characterized by anovulation and hyperandrogenism; (3) phenotype 3, characterized by hyperandrogenism and polycystic ovaries; and (4) phenotype 4, characterized by anovulation and polycystic ovaries. Difference in the risks of metabolic disorders were also observed between phenotypes. Similarly, insulin resistance profiles are also found to be different among phenotypes. The most severe form of PCOS is phenotype 1, with higher level of AMH compared to other phenotypes and normal population. However, there are no clear correlations between levels of AMH with the incidence of insulin resistance in PCOS patients. One study involving non-obese Chinese women found AMH as a useful parameter to assess different phenotypes of PCOS using HOMA-IR marker found no correlation between phenotypes and insulin resistance [14], but similar study has never been conducted on Indonesian population. Thus, the present study tries to look at the relationship between AMH and HOMA-IR in different phenotypes of PCOS especially in the Indonesian population.

## Methods

### Study design

This is a cross-sectional study involving reproductive age women between the age of 18 and 35 years old who came to Yasmin Clinic, Dr. Cipto Mangunkusumo General Hospital, Jakarta, Indonesia between 2011 and 2014. One inclusion criterion is PCOS woman diagnosed using Rotterdam criteria [13], in which diagnosis are established if the patient fulfilled two out of three of the following criteria: (1) oligo-/anovulation, (2) hyperandrogenism (clinically and/or biochemically), (3) polycystic appearance on ultrasound. Another inclusion criterion is serum AMH levels above 4.45 ng/mL. Patients with a history of laparoscopic ovarian drilling and use of insulin sensitizer were excluded from this study.

Ethical approval has been obtained from the Human Ethics Committee of Faculty of Medicine, University of Indonesia. After informed consent was obtained, all patients underwent a general clinical examination.

### Measurements and laboratory analyses

Fasting insulin, AMH and Homeostatic Model Assessment of Insulin Resistance (HOMA-IR) measurements were performed on the serum of each subjects. Level of AMH will be examined using a Gen 2 ELISA kit (Beckman Coulter, Inc. Brea, CA, USA).

Hyperandrogenism is defined as free androgen index (FAI) > 5; FAI is calculated as testosterone level (nmol/L) divided by SHBG level [15]. Total testosterone, fasting glucose, fasting insulin, and serum FSH, luteinizing hormone (LH), and prolactin levels were also examined.

Insulin resistance was measured using the Homeostatic Model Assessment of Insulin Resistance (HOMA-IR) with the following formula: fasting insulin (mIU/L) × fasting plasma glucose (mg/dL)/405 [16].

### Statistical analysis

Kolmogorov–Smirnov tests were used to assess the normality of data. Normally distributed numerical data are presented as mean ± standard deviation, and numerical data not normally distributed are presented as median (range). Kruskal–Wallis test was used to compare the results between the 4 phenotypes; post hoc analyses were conducted using independent *t* tests or Mann–Whitney tests for normally-distributed and non-normally-distributed data, respectively. Pearson or Spearman correlations were performed with HOMA-IR as the dependent variables. All parameters with a p value < 0.25 in the correlation analyses were then included in a multivariate linear regression analysis, using a backward elimination method, to determine the variables correlated with the HOMA IR value. Statistical analysis was performed using SPSS for Windows version 11.0 (SPSS Corp, Chicago, IL).

## Results

### Subject characteristics

A total of 125 patients were included in this study. The mean age of patients was 29.6 ± 4.5 years and the mean BMI was 25.7 ± 4.8 kg/m^2^; with around half of the patients (63 patients, 50.4%) were obese (Table 1). Phenotype 1 was found to be the most common phenotype among all four phenotypes (n = 39, 31.2%), followed by phenotype 4 (Table 2).

**Table 1:** Characteristics of the subjects with polycystic ovary syndrome

Characteristics	Phenotype			
	1 (n = 39)	2 (n = 27)	3 (n = 26)	4 (n = 33)
Age (years)	29 (20–39)	29 (19–35)	32 (23–39)	28 (21–36)
BMI (kg/m^2^)	25.5 ± 4.8	25.8 ± 3.5	25.7 ± 5.0	26.0 ± 5.7
% body fat	31.8 (19.2–50.7)	32.1 (25.1–45.5)	29.3 (23.7–45.8)	34.9 (21.1–60.8)

BMI are reported as mean ± standard deviation

Age and % body fat—values are reported as median (range) since the data distribution is not normal

**Table 2:** Distribution of the patients among the four polycystic ovarian syndrome phenotypes (n = 125)

Phenotype	OA	HA	PCO	n	%
1	+	+	+	39	31.2
2	+	+	–	27	21.6
3	–	+	+	26	20.8
4	+	–	+	33	26.4

*OA* anovulation, *HA* hyperandrogenism, *PCO* polycystic ovaries

### Fasting insulin and plasma glucose levels

**Fig. 1:**
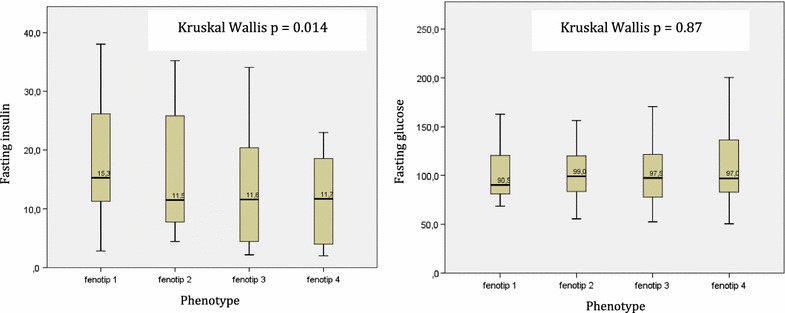
Fasting serum insulin (mIU/L) and glucose (mg/dL) levels in patients with polycystic ovarian syndrome

The mean fasting plasma glucose and insulin levels for the entire sample were 103.6 ± 29.1 mg/dL and 14.9 ± 9.6 mIU/L, respectively (Fig. 1). There was a significant difference in fasting insulin levels among the four phenotypes (p = 0.014) based on the Kruskal–Wallis test.

### Hormonal characteristics

The median AMH level was 8.80 (3.10–23.82) ng/mL. Phenotype 1 shows significantly higher AMH levels (Kruskal–Wallis, p < 0.05) than other phenotypes. Post-hoc analyses showed significant differences between phenotypes 1 and 3, phenotypes 1 and 4, and phenotypes 2 and 4. Serum LH levels also tended to be higher with phenotype 1. However, there are no differences in FSH level among the four phenotypes. LH levels and LH/FSH ratio were found to be correlated with serum AMH levels (*r* = 0.58 p < 0.001 and *r* = 0.40, p < 0.001, respectively) (Table 3).

**Table 3:** Hormonal characteristics based on the Rotterdam criteria for polycystic ovarian syndrome

Hormone levels	Phenotype			
	1 (n = 39)	2 (n = 27)	3 (n = 26)	4 (n = 33)
AMH (ng/mL)	11.7 (4.5–23.8)	9.9 (4.6–19.5)	7.5 (4.6–23.8)	7.1 (3.1–9.6)
LH (mIU/mL)	8.7 (5.0–18.8)	8.6 (2.0–23.1)	5.1 (4.1–13.5)	5.4 (2.0–9.9)
FSH (mIU/mL)	4.7 (0.4–7.7)	4.0 (1.1–6.5)	4.8 (0.4–21.2)	5.5 (1.7–9.6)
LH/FSH ratio	2.1 (0.9–32.0)	2.4 (0.3–10.6)	1.2 (0.5–10.3)	1.1 (0.5–4.0)
HOMA IR	4.2 (0.5–8.2)	3.2 (0.7–8.1)	2.9 (0.6–8.5)	2.1 (0.4–4.9)

Values are reported as median (range)

We found HOMA-IR to be correlated with BMI (*r* = 0.19, p = 0.04), LH level (*r* = 0.27, p = 0.002), LH/FSH ratio (*r* = − 0.20, p = 0.03), and AMH (*r* = 0.52, p < 0.001). Thus, BMI, LH/FSH ratio, and AMH were included in the multivariate linear regression analysis (Table 4). The multivariate analysis generated an equation for HOMA IR as follows: − 3.61 + 0.08 (BMI) + 5.33 (log AMH) with an adjusted R^2^ = 0.30.

**Table 4:** Linear regression model for the homeostatic model assessment for insulin resistance (HOMA IR)

Variables	Unstandardized B (standard error)	Standardized B	p
BMI	0.08 (0.03)	0.19	0.015
AMH^a^	5.33 (0.76)	0.53	< 0.001
Constant	− 3.61 (1.09)		0.001

Adjusted R^2^ = 0.30

*BMI* body mass index, *AMH* anti-mullerian hormone

^a^Log-transformed

## Discussion

This is the first study looking at the relationship between AMH and HOMA-IR in different types of PCOS phenotypes, especially in Indonesian population. The most prevalent phenotype of PCOS in the present study was phenotype 1 (anovulation, hyperandrogenism, and polycystic ovaries) in which consistent with a previous study showing 46% of PCOS patients were found to have phenotype 1 [17]. Furthermore, it was found that the more severe the phenotype of PCOS, the higher the level of HOMA-IR. However, this study finds equal distribution among for all four phenotypes, this might be due to the low prevalence of hyperandrogenism in Asian population compared with other races [18, 19].

LH levels and the LH/FSH ratio were highest with phenotype 1 and lowest with phenotype 2, which was similar to the findings of a study conducted in China [20]. A relative FSH deficiency might stimulate the secretion of LH in patients with PCOS [15]. Moreover, a study conducted in an Iran population revealed high LH levels in phenotype 1 patients corresponding to high levels of both testosterone and androgen [16].

Level of AMH has also been studied to have correlation with oligo-/anovulation [21] and the level of AMH were 12 times higher among anovulatory PCOS patients with PCOS compared to ovulatory PCOS patients [22].

The mean age of PCOS patients in the present study is 29.6 years old, this is believed to due to a declining number of antral follicles as the patients age advance [23, 24]. However, present study found no significant differences of age group among patients with different PCOS phenotypes.

One of the proposed underlying pathophysiology of PCOS is insulin resistance. It is estimated that around 50–80% of patients with PCOS also has insulin resistance. In the current study, we found an association between AMH and IR (*r* = 0.52, p < 0.001). Although this relationship remains controversial, with some researchers reporting positive association while others reporting no association, the present study shows very significant correlations between AMH level and IR. Insulin is a reproductive and metabolic hormone. An increase in insulin levels will augment androgen production in PCOS [12].

To date, there is no agreement on which is the best method to evaluate the incidence of insulin resistance in PCOS. Many publications proposed the use of HOMA-IR as a standard measurement for insulin resistance diagnosis [25]. The level of HOMA-IR was found at its highest level among PCOS patients with phenotype 1. The level decreases in accordance with the severity of PCOS. Although BMI did not differ by phenotype, the differences in AMH levels and HOMA-IR tend to follow the spectrum of the four phenotypes. Higher HOMA IR has also been reported in phenotype 1 compared to other phenotypes as well as controls [20]. Fasting insulin level significantly differs between all phenotypes (Fig. 1), however, consistent with a previous study, we found no difference in fasting plasma glucose levels [16]. Thus, patients with PCOS might have abnormal insulin levels despite having normal plasma glucose levels. Although current guidelines focus on plasma glucose to diagnose diabetes in the general population [26], physicians treating patients with PCOS should not rely only on normal glucose levels. Moreover, these findings might support the benefit of metformin therapy to lower androgen levels in patients with PCOS [27].

A significant positive correlation between AMH and HOMA-IR was observed in the current study, despite the fact that a causative relationship could not be determined. This possible link between the two hormones has been discussed among healthy women population [28]. Skalba, et al. found a moderate positive correlation between AMH and HOMA-IR (*r* = 0.62, p < 0.001) [29]. However, a negative correlation between AMH and HOMA-IR has also been reported [9].

This study has several limitations. Owing to its cross-sectional design, a causative relationship between AMH and insulin resistance could not be explored. Data regarding standardized screening of diabetes is also limited, and previous history of diabetes mellitus were not obtained. Furthermore, because patients were recruited from a tertiary hospital, previous medication history could not be fully obtained. Additionally, other variables related to insulin resistance (e.g., physical activity and diet history) could not be quantified. The correlation between the AMH and HOMA-IR values should be interpreted with caution, as AMH might be a predictor of insulin resistance. To our knowledge, no study has reported direct relationship between AMH and insulin resistance. Due to a lack of data regarding androgen levels, the results might be overestimate as AMH is also reported to be positively associated with serum androgen levels [30].

## Conclusions

There was a positive correlation between serum AMH and HOMA-IR levels. Serum AMH and HOMA-IR levels were significantly different across the four PCOS phenotypes; with the highest values observed in patients with phenotype 1.

## Abbreviations

PCOS: polycystic ovarian syndrome
AMH: anti-mullerian hormone
HOMA-IR: homeostatic model assessment for insulin resistance
SHBG: sex hormone binding globulin
BMI: body mass index
FSH: follicle stimulating hormone
LH: luteinizing hormone
ELISA: enzyme linked immunoabsorbent assay
TGF-β: transforming growth factor β
